# Genome-wide identification and expression analysis of the *NHX* gene family under salt stress in wheat (*Triticum aestivum* L)

**DOI:** 10.3389/fpls.2023.1266699

**Published:** 2023-12-04

**Authors:** Pradeep Sharma, Shefali Mishra, Bharati Pandey, Gyanendra Singh

**Affiliations:** ^1^ Crop Improvement division, ICAR-Indian Institute of Wheat and Barley Researh, Karnal, India; ^2^ Division of AgriBioinformatics, ICAR-Indian Agricultural Statistics Research Institute, New Delhi, India

**Keywords:** Na +/H + antiporter, amiloride-binding site, molecular dynamics, gene expression, salt stress, wheat

## Abstract

Salt stress affects plant growth and development, resulting in the loss of crop yield across the world, and sodium-proton antiporters (NHXs) are one of the genes known to promote salt tolerance in transgenic plants. In this study, we conducted a comprehensive genome-wide analysis and expression profile of *NHX* genes in wheat under salinity stress. We identified 30 *TaNHX* genes in wheat based on the Na^+^/H^+^ exchanger domain, with all genes containing an amiloride motif except one, a known for inhibiting Na^+^ ions in plants. Phylogenetic analysis classified these genes into three classes with subfamilies: 12 were localized in vacuoles, while 18 were in the endoplasmic reticulum and plasma membrane. Promoter analysis revealed stress-related *cis*-acting elements, indicating their potential role in abiotic stress tolerance. The non-synonymous (K_a_)/synonymous (K_s_) ratios highlighted that the majority of *TaNHX* genes experienced robust purifying selection throughout their evolutionary history. Transcriptomis data analysis and qRT-PCR demonstrated distinct expression patterns for *TaNHX* genes across various tissues when subjected to salt stress. Additionally, we predicted 20 different miRNA candidates targeting the identified *TaNHX* genes. Protein-protein interaction prediction revealed NHX6’s involvement in the SOS1 pathway, while *NHX1* gene exhibit proton antiporter activity. Molecular dynamics (MD) simulations were also conducted to examine the interactions of *TaNHX1*, *TaNHX2*, and *TaNHX3*. These results represent a significant advancement in our understanding of the molecular mechanisms governing Na^+^ transporters. This may also offer promising avenues for future studies aimed at unraveling the intricate details of their biological roles and applications.

## 1 Introduction

Soil salinity stands as a formidable challenge with far-reaching consequences for agriculture on a global scale, impacting a staggering 45 million hectares of irrigated land (Wu et al., 2019). This issue carries immense significance, as it directly influences the capacity to sustain agricultural productivity. This becomes particularly critical when we consider that irrigated lands, renowned for their ability to yield twice the food production compared to rain-fed areas (Aharon et al., 2003), are pivotal in addressing the pressing concerns of food security and resource sustainability. Furthermore, projections indicate that around 50% of cultivable land could be affected by excessive salinization by 2050 (Gaxiola et al., 1999; Pehlivan et al., 2016). The increasing global population further underscores the need to resolve this issue to secure food security and the sustainability of agricultural practices.

In response to the adverse effects of salt stress, plants activate a range of sophisticated mechanisms to ensure their survival. These include complex hormonal regulation of plant growth and metabolism, precise osmotic regulation, and ion homeostasis maintenance (Wu et al., 2019a). The role of sodium/hydrogen antiporter (NHX) proteins is pivotal in regulating ion homeostasis. These proteins intricately modulate the equilibrium of ions, as underscored by their interplay with the essential proton pumps, the H+-PPase and H+-ATPase enzymes. Together, they meticulously orchestrate the translocation of sodium ions (Na+) from the dynamic cytoplasmic milieu to designated cellular destinations, including vacuolar compartments and extracellular regions. This ion flux operation serves as a vigilant guardian, staunchly preventing the potential deleterious accrual of Na^+^ ions within the intricate confines of cellular compartments (Aharon et al., 2003; Dragwidge et al., 2019).

The *TaNHX* gene family encodes widely distributed transmembrane proteins classified under the monovalent cation/proton antiporter 1 (CPA1) category (Fukuda et al., 2011). Prior studies (Wu et al., 2019a) have revealed that NHX proteins typically encompass 10–12 transmembrane helices (TMs) and predominantly inhabit vacuolar, endosomal, and plasma membrane locales (Aharon et al., 2003; Pehlivan et al., 2016). In *A. thaliana*, eight distinct NHX genes were identified (Gaxiola et al., 1999). These genes exhibit diverse subcellular distributions, with two *NHX* genes (AtNHX7 and AtNHX8) residing within the plasma membrane (PM-class), four inhabiting vacuoles (Vac-class), and two localizing to endosomes (Endo-class) (Shi et al., 2000; Aharon et al., 2003; Brett et al., 2005; Bassil et al., 2011). NHX proteins are distributed in a way that they reflects the specific roles they play in maintaining ion homeostasis in different environmental conditions.

Understanding the different mechanisms of *NHX* gene function is critical for decoding the plants complex response to salt stress. We can gain a valuable understanding of the critical roles that *TaNHX* genes play in regulating salt stress in various plant species by examining their regulatory functions, and evolutionary relationships. Such bioinformatics studies and experimental investigations pave the way for future research and manipulation of *NHX* genes, contributing to the production of salt-tolerant crop varieties and enhancing agricultural productivity in salt-affected regions.

*NHX* genes are intricately involved in a multitude of biological processes, including response to salt stress, regulation of cell growth, modulation of membrane vesicle trafficking, and maintenance of pH homeostasis (Dragwidge et al., 2019). After inactivating the mutation in *AtNHX5* and *AtNHX6*, it was observed that there were development-related disorders and anomalies in cell division in tissues of the embryo and roots (Dragwidge et al., 2019). *OsNHX1, OsNHX2, OsNHX3*, and *OsNHX5* in rice become activated when they are exposed to salt stress, hyperosmotic stress, and ABA stress conditions. Determining the salt tolerance mechanisms within the rice plant may be delineated by the nuanced expression patterns of *NHX* genes across diverse tissue types (Fukuda et al., 2011). In other experiments, rye grass engineered with the rice vacuolar membrane *OsNHX1* gene endured high salt conditions (350 mM) for 10 weeks, while transgenic *B. napus* plants thrived even in the presence of 200 mM NaCl. Introducing the Vac-class membrane gene *AgNHX1* from *A. gmelinii* into rice plants conferred the ability to withstand 300 mM NaCl for three days, surpassing the salt sensitivity of wild-type rice. Overexpression of *AtNHX1* from *A. thaliana* into tomato and *SsNHX1* from *Salsola collina* into *M. sativa* significantly improves salt tolerance in plants. These findings underscore the potential of *NHX* genes to enhance salt tolerance in various plant species (Manik et al., 2015; Wilkins et al., 2016; Kumari et al., 2018; Wu et al., 2019a). The most widely grown crop in the world is wheat. Its growth is significantly impeded by salt stress since it is not a halophyte. When exposed to salt, wheat cells quickly increase their Na^+^ concentration. Due to the relatively low net uptake compared to unidirectional inflow (Khare et al., 2015), it was expected that wheat roots would experience high rates of Na^+^ efflux. The ability of a wheat Na^+^/H^+^ antiporter (TaSOS1) to extrude Na^+^ across the plasma membrane has been experimentally studied (Parveen et al., 2021). Overexpression of the vacuolar NHX antiporter *AtNHX1* from Arabidopsis led to an increase in wheat salt tolerance (Parveen et al., 2021). The putative wheat Na^+^/H^+^ antiporter TaNHX1 provided salt tolerance to transgenic Arabidopsis plants (Ramesh et al., 2019). In a previous study, *TaNHX2* was cloned from bread wheat, and it was examined for its expression and function of the protein and yeast. The proposition that *TaNHX2* mediates the compartmentalization of Na^+^ into vacuoles holds promise for elucidating its pivotal role in enhancing a plant’s salt tolerance. Nevertheless, the precise mechanisms underlying TaNHX2’s participation in Na^+^/H^+^ exchange and its subcellular localization within plant cells remain unverified (Shuai et al., 2013). In this study, we performed a genome-wide investigation of all *TaNHX* gene family members in wheat. The *TaNHX* genes were identified using gene structure, chromosomal locations, phylogenetic relationships, *cis-*element, and expression profiling, as well as molecular dynamic simulations. The expression patterns obtained from *in-silico* analysis were then confirmed using qRT-PCR. *TaNHX* can be used to breed salt tolerance using genome-editing methods. The results taken here are necessary to systematically explore the gene function of the wheat *TaNHX* gene family.

## 2 Materials and methods

### 2.1 Identification, characterization, phylogenetic analysis and gene ontology of sodium proton antiporters

To identify *TaNHX* genes in wheat, we employed a bioinformatics approach. Firstly, we obtained protein sequences of NHX from Arabidopsis, cotton, sorghum, and barley, which served as query sequences. These query sequences were then searched against the wheat genome available at the Ensembl Genomes database (fp://fp.ensemblgenomes.org/pub/plants/release-51/fasta/triticumaestivum/pep/) using the BLASTP algorithm. We selected all homologous protein sequences of NHX candidates that met the specified criteria, including an e-value threshold of 1e-10 and a bit score value higher than 100 percent. To further validate the presence of the Na^+^_H^+^_Exchanger domain (PF00999) characteristic of NHX transporters, the obtained sequences were scanned against this domain using the HMMER 3.1b2 online software (https://www.ebi.ac.uk/Tools/hmmer/) (Potter et al., 2018).

For the subcellular localization, Wolf Psort (https://wolfpsort.hgc.jp/) was used. The physio-chemical properties were predicted using the online programme ProtParam (http://web.expasy.org/protparam/).To study the evolutionary linkage, Clustal W software was used to analyze NHX protein sequences from Arabidopsis, barley, sorghum, cotton, and wheat (Larkin et al., 2007). Following that, an unrooted phylogenetic tree was built in MEGA-X with 1000 bootstrap repetitions using Maximum likelihood (ML) with default parameters the JTT model with uniform rates and 4 number of threads (Kumar et al., 2018). NHX members in wheat were classified into subfamilies based on their Arabidopsis, barley, sorghum, and cotton homologs.

Functional annotation of the identified genes was performed using Blast2GO (https://www.blast2go.com/). This analysis enabled the elucidation of molecular functions, biological processes, and cellular components intricately linked to the genes under investigation. Additionally, we harnessed the KEGG (Kyoto Encyclopaedia of Genes and Genomes) database (http://www.genome.jp/kegg/) to annotate the metabolic pathways, thus furnishing invaluable insights into the precise pathways where these genes actively participate. The combination of Blast2GO and KEGG analysis allowed for a comprehensive understanding of the functional properties and metabolic roles of the identified genes.

### 2.2 Conserved motifs, gene structure, promoter analysis and physical mapping of NHX genes

To unravel the presence of conserved motifs within the identified genes, we used the MEME webserver (Multiple Em for Motif Elicitation). Employing default parameters, which encompassed the exploration of 2-20 motif sites, the extraction of up to 10 motifs, and a motif width range extending from 6 to 50 (Bailey et al., 2009), this analysis provided an intricate glimpse into the genetic signatures. Furthermore, we delved into the gene structure using the Gene Structure Display Server (GSDS) tool (Hu et al., 2015), unravelling the intricacies of these genes’ architectural features. This comprehensive approach not only unveiled conserved motifs but also shed light on the structural nuances inherent to the genes under study. The promoter sequences, of up to 2 kb in size, were used for *cis*-acting element analysis and submitted to the PLANTCARE webserver. The *cis-*acting elements that were produced were categorized according to their functional class.

Based on the position sites, the chromosomal locations of the wheat *TaNHX* genes were examined. The Ensembl database is used to retrieve information about the Ta*NHX* gene’s locations. Physical mapping of the transporters was constructed using Map-Chart software to construct the location on the wheat genome (Voorrips, 2002).

### 2.3 Protein-protein interaction, miRNA targeting, and gene duplication events

For the identification of functional protein-protein interactions, the STRING v1054 databases were employed (Szklarczyk et al., 2015). However, Arabidopsis was used as the reference species to search the interactive network in the database. Blast the sequences with set parameters to an e-value of 1e-10, and the *A. thaliana* genome was searched against all known interaction partners. The best-hit gene for each gene was chosen using Cytoscape 58 to create a PPI network. Using Cytoscape’s cyto Hubba plugin, the top hub gene from the interaction network was finally determined. Furthermore, we conducted an analysis to pinpoint significant Gene Ontology (GO) terms (FDR ≤ 0.01) related to the molecular functions and biological processes of the interaction network nodes using the iDEP webtool (Ge et al., 2018). The transcript sequences of the *TaNHX* gene were obtained using the Ensembl plant database. Now, the transcript sequences of *TaNHX* and the mature miRNA sequences from miRbase (Kozomara and Griffiths-Jones, 2014) were analyzed by using the psRNATarget service’s default settings (Dai and Zhao, 2011) to find the targets of miRNAs.

We accessed the coding sequences (CDS) of *T. urartu* (A genome), *A. tauschii* (D genome), and *T. dicoccoides* (AB genome) from Ensembl plants (fp://fp.ensemblgenomes.org/- pub/plants/release-42/fasta/triticum_urartu,fp://fp.ensemblgenomes.org/pub/release-42/plants/fasta/aegilops_tauschii/cds,fp://fp.ensemblgenomes.org/pub/release-42/plants/fasta/triticum_dicoccoides/cds/). These genomes served as references for the A, D, and AB genomes, respectively. We conducted BLASTn searches using these sequences against the TaNHX CDS sequences in *T. aestivum* to identify orthologous genes. Top hits were designated as orthologs within each species, adhering to stringent criteria, including an e-value cut-off of 1e-10 and a 150-bit-score cut-off. This approach was similarly applied to search for orthologous genes in other monocots and dicots, including *O. sativa*, *H. vulgare*, *Z. mays*, and *A. thaliana*. To examine the synteny relationships of the *NHX* gene family within and across various species, we employed TBtools for a comprehensive analysis.

### 2.4 Gene expression profiling using RNA-seq data

To determine *in-silico* gene expression in different tissues under salinity stress, the log_2_ value of the FPKM value was calculated using the SRA data (SRP304900) from NCBI. Heatmaps were created with Clustvis (https://biit.cs.ut.ee/clustvis/).

### 2.5 Sample collection and treatments

To investigate the expression patterns of *TaNHX* genes in response to salinity stress, we sourced wheat genotypes, specifically KRL213 and HD2009, from the Germplasm Unit of the ICAR-Indian Institute of Wheat and Barley Research, located in Karnal, India. In a controlled environment, we initiated the germination process by placing the seeds in Petri dishes at a temperature of 22°C. Prior to germination, the seeds were sterilization using a 1% sodium hypochlorite solution for duration of 10 minutes, followed by thorough rinsing with distilled water, ensuring the removal of any residual sterilization agents. After five days of germination, seedlings were transferred to full-strength Hoagland’s solution phytojars and incubated for 14 days in a BOD incubator with two sets of three biological replicates of each genotype. For salt stress, two contrasting wheat genotypes, HD2009 (salt sensitive) and KRL213 (salt tolerant), were used. Both genotypes at the two-leaf seedling stage were stressed with 150 mM NaCl. After the treatment, the leaf samples were taken at 0, 3, 24, and 48 hours. For total RNA isolation, all acquired samples were immediately wrapped in foil and frozen in liquid nitrogen at -80°C.

### 2.6 RNA extraction and qRT-PCR analysis

To isolate RNA, we employed TRIzol reagent following the manufacturer’s guidelines. The extracted RNA was further processed to eliminate any residual DNA through DNase I treatment (NEB, USA). Utilizing Superscript-III reverse transcriptase (Invitrogen, USA), 1 µg of total RNA was converted into the first strand of cDNA. This resulting cDNA was subsequently diluted at 1:2 ratio, and 1 µl of the diluted cDNA was employed as a template within a 10 µl reaction volume for real-time qRT-PCR analysis. We conducted SYBR Green-based real-time quantitative RT-PCR analysis using the BIO-RAD CFX96 system (Bio-Rad). For normalization purposes, we employed wheat actin as an endogenous control (Muthusamy et al., 2016). The expression levels were quantified as relative fold changes through the application of the 2^△△-Ct^ method (Livak and Schmittgen, 2001). All the samples were set up in 3 biological replicates. Experimental data was statistically analyzed by a one-way Analysis of Variance (ANOVA) and Tukey’s multiple range test was used for mean value separation by PAST software (Hammer et al., 2001). P-value of <0.05 was considered statistically significant.

### 2.7 Molecular modelling and dynamics simulations

The three-dimensional structure for TaNHX was generated using AlphaFold2 (Skolnick et al., 2021), as it employs deep learning techniques, specifically deep neural networks, to predict protein structures with remarkable accuracy. It leverages vast amounts of protein sequence and structural data to generate predictions of protein structures, even for proteins with no known structures.

The molecular dynamics simulations (MDS) were performed using GROMCAS 5.0 (Abraham et al., 2015). The simulations aimed to investigate the conformational changes of the TaNHX protein structures in the presence of a solvent system. To set up the simulations, the OPLS_2005 force field (Dubay et al., 2012) was employed to describe the interactions within the proteins. The system was solvated in a cubic water box using the SPC water model. The protein atoms were kept at a minimum distance of 10 Å from the edges of the box. To achieve a neutral charge for the systems, counter ions were added, and a 0.15 M ionic concentration was maintained by including Na^+^ and Cl ions. First, each system underwent 50,000 steps of steepest descent energy minimization, which helped eliminate steric overlap and stabilize the initial configurations. Following the energy minimization, a two-step equilibration phase was performed. The first step was NVT equilibration, where the number of particles, volume, and temperature were kept constant. This phase ran for 100 picoseconds (ps) to stabilize the system’s temperature. The V-rescale temperature-coupling method was used for NVT, with a constant coupling time of 1 ps and a target temperature of 303.15 K. The second step was NPT equilibration, where the number of particles, pressure, and temperature were maintained constant. This phase also ran for 100 ps, and it involved using the Nose-Hoover pressure coupling method with a constant coupling time of 1 ps and a target temperature of 303.15 K. During this phase, the system was relaxed, and the protein was restrained using position restraints (h-bonds). To account for electrostatic interactions, the Particle Mesh Ewald method was employed for both NVT and NPT simulations. After the equilibration phases, each system underwent a full production run of 30 nanoseconds (ns) without any restraints. The integration time step used was 0.002 ps, and coordinates were recorded every 10 ps using an xtc collection interval of 5,000 steps.

## 3 Results

### 3.1 Characterization, phylogenetic analysis, and gene ontology of TaNHX genes in wheat

In order to identify members of the *TaNHX* gene family within the wheat genome, we employed a two-step approach. Firstly, utilized known NHX sequences from other species as queries and performed a blast search against the wheat proteome database (**Supplementary Table S1**). This enabled us to retrieve putative *TaNHX* genes in wheat. Subsequently, we eliminated redundant sequences and confirmed the gene identifications based on the presence of the Na^+_^H^+^_Exchanger domain (PF00999). This stringent filtering process resulted in the identification of 30 *TaNHX* genes in *T. aestivum*.

To understand the physio-chemical properties of the wheat *TaNHX* genes, we conducted a comprehensive analysis, the results of which are summarized in **Table 1**. Hence, to determine the subcellular localization of NHX proteins, prediction tools have been employed, which were found to predominantly reside on the plasma membrane, endosomes, and vacuoles. Furthermore, we assessed several parameters, including the number of amino acids, which ranged from 374 to 1191, reflecting the diversity in NHX protein lengths. Additionally, the molecular weight of the NHX proteins revealed a wide range of values. For instance, TaNHX16 exhibited a molecular weight of 40.84 kDa, whereas TaNHX11 displayed the highest molecular weight of 131.42 kDa. The findings presented in **Table 1** shed light on the distinct physiochemical characteristics of the identified wheat *NHX* genes.

**Table 1:** Features of the 30 NHX proteins from *T. aestivum* identified in this study.

Gene Name	Transcript ID	Chr. No.	Start	End	No. of aa	Mole.weight	pI	Inst.Index	Aliphatic index	GRAVY	Sub-cellul. Loc.
TaNHX1	TraesCS1A02G102300.1	1A	98242495	98247975	546	59704.39	8.13	31.03	117.66	0.675	plas
TaNHX2	TraesCS1B02G112700.1	1B	130636812	130643984	1034	114574.21	5.92	44.44	100.93	0.074	plas
TaNHX3	TraesCS1D02G093900.1	1D	79434999	79440799	546	59718.42	8.13	31.26	117.84	0.679	plas
TaNHX4	TraesCS2A02G034700.1	2A	15185525	15223170	546	59712.37	8.14	31.01	117.66	0.68	plas
TaNHX5	TraesCS2A02G121000.1	2A	70876938	70881306	538	59112.45	8.41	33.39	112.17	0.591	plas
TaNHX6	TraesCS2A02G133600.1	2A	80270608	80279857	760	84097.17	6.52	31.25	110.76	0.321	plas
TaNHX7	TraesCS2B02G141900.1	2B	108237682	108242312	598	66390.72	9.2	41.21	105.97	0.393	plas
TaNHX8	TraesCS2D02G123000.1	2D	71767337	71771197	532	58542.8	8.13	32.62	112.71	0.604	plas
TaNHX9	TraesCS2D02G135600.1	2D	79692600	79701662	771	85272.1	6.56	33.29	110.45	0.337	plas
TaNHX10	TraesCS3A02G023200.2	3A	12969505	12980909	1142	126220.06	6.87	43.19	104.84	0.113	plas
TaNHX11	TraesCS3B02G021600.2	3B	9165564	9177438	1191	131426.28	8.52	45.66	103.16	0.085	plas
TaNHX12	TraesCS3D02G022900.1	3D	7256680	7268617	1137	125621.34	6.87	43.91	104.71	0.107	plas
TaNHX13	TraesCS4A02G145300.1	4A	246708879	246714419	540	59245.5	8.52	34.99	111.22	0.549	vacu:
TaNHX14	TraesCS4B02G125700.1	4B	156526868	156534017	416	45825.48	8.72	38.44	109.69	0.438	plas
TaNHX15	TraesCS4D02G147600.1	4D	139042047	139047298	477	52617.06	8.53	30.19	119.39	0.692	vacu /Plas
TaNHX16	TraesCS5A02G176100.1	5A	370239620	370247353	374	40846.21	5.06	47.58	115.75	0.866	plas
TaNHX17	TraesCS5A02G260700.1	5A	474579384	474586850	532	58648.64	5.12	45.41	103.42	0.409	plas
TaNHX18	TraesCS5B02G173800.2	5B	318992910	319002092	374	40862.21	5.06	48.09	115.48	0.859	plas/vac
TaNHX19	TraesCS5B02G259100.1	5B	441532328	441539514	522	57654.52	5.1	45.1	104.1	0.443	plas
TaNHX20	TraesCS5D02G180800.1	5D	281021983	281031600	534	58431.65	5.03	47.01	102.45	0.46	plas
TaNHX21	TraesCS5D02G268200.1	5D	371663980	371671626	532	58789.86	5.2	44.96	103.97	0.406	plas
TaNHX22	TraesCS7A02G228400.1	7A	198804283	198808214	527	58011	8.61	33.71	108.39	0.61	vacu
TaNHX23	TraesCS7A02G242300.1	7A	217580011	217587424	542	59354.31	5.29	48.14	103.3	0.347	plas
TaNHX24	TraesCS7A02G397700.2	7A	576440908	576454559	998	109881.16	5.77	34.71	105.64	0.207	plas
TaNHX25	TraesCS7A02G462000.1	7A	658637042	658651135	991	109173.5	5.9	36.22	107.37	0.219	plas
TaNHX26	TraesCS7B02G149100.2	7B	196868440	196874596	537	58832.88	5.37	46.55	105.53	0.429	plas
TaNHX27	TraesCS7B02G191300.1	7B	329030544	329034430	527	58145.11	8.76	34.19	106.36	0.572	vacu
TaNHX28	TraesCS7B02G475500.1	7B	731431027	731444592	1141	126185.71	6.25	42.41	101.52	0.082	plas
TaNHX29	TraesCS7D02G226200.1	7D	186316884	186320911	527	58058.03	8.61	33.95	107.1	0.593	vacu
TaNHX30	TraesCS7D02G241200.1	7D	205547583	205554255	543	59569.54	5.31	49.17	104.18	0.352	plas

**Figure 1:**
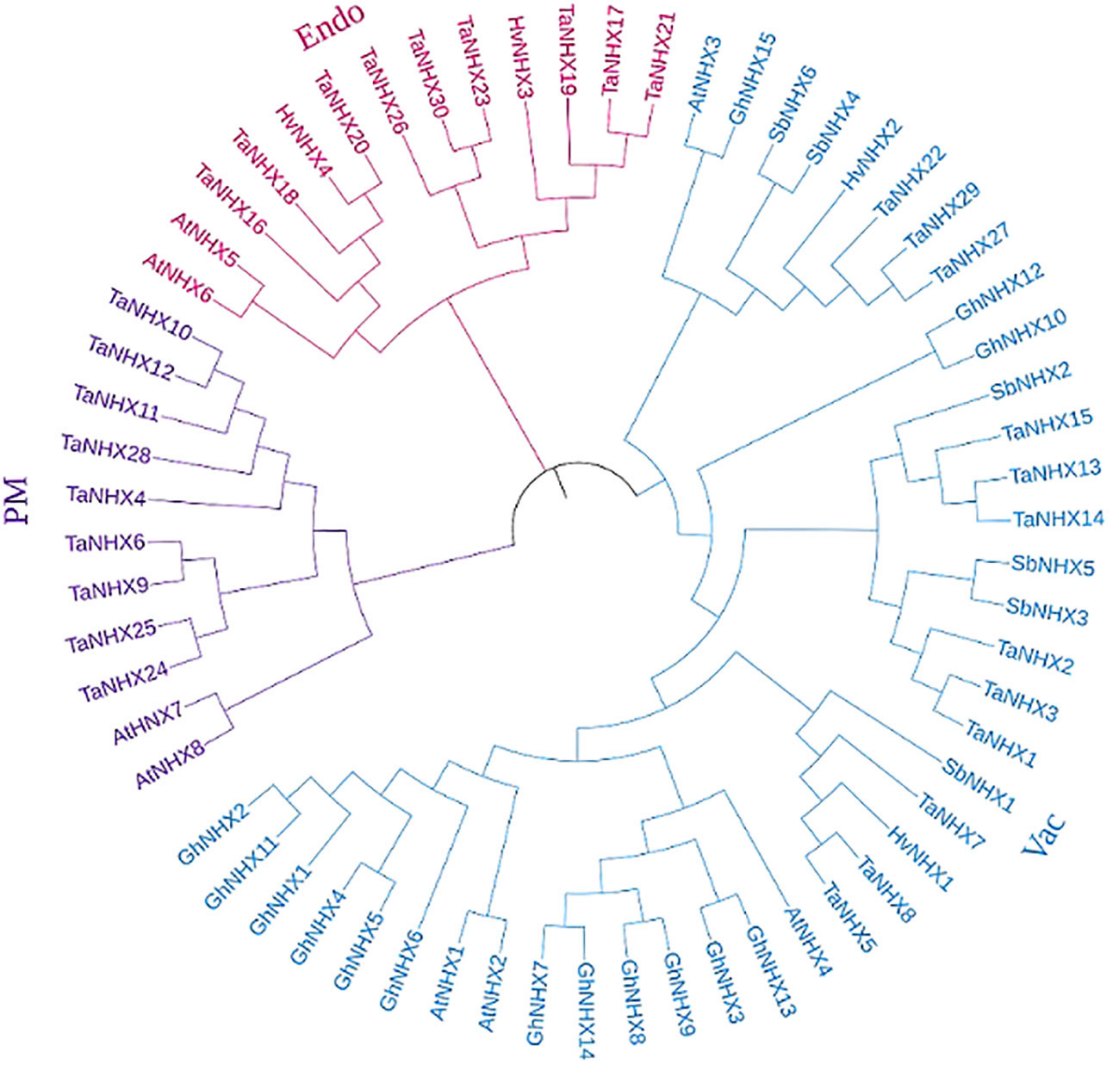
Phylogenetic trees showing relationships of *NHX* genes family of *T. aestivum, A. thaliana, S. bicolor, H. vulgare, and G. hirusitum*. The trees were constructed using the maximum likelihood method and a bootstrap replicate of 1000. The trees with the highest bootstrap support for each gene class have been shown here. The three major classes (Vac-, Endo-, and PM-) are marked with different colors.

To analyze the evolutionary lineages of the *NHX* gene family across different species, a dataset comprising 63 NHX protein sequences was utilized. The sequences were obtained from five species, namely *A. thaliana, H. vulgare, G. hirsutum, S. bicolor*, and *T. aestivum* (**Supplementary Table S2**). These sequences were employed to construct a phylogenetic tree, as depicted in **Figure 1**. The analysis involved the utilization of previously reported genes, resulting in the classification of the 63 genes into three distinct categories: class I (endo-class), class II (PM-class), and class III (vac-class). Among the five species, the largest group was class III (vac-class), which encompassed 39 proteins. Specifically, this group comprised 4 NHX proteins in *A. thaliana*, 2 in *H. vulgare*, 15 in *G. hirsutum*, 6 in *S. bicolor*, and 12 in *T. aestivum*. It is worth noting that 9 TaNHX proteins were distributed evenly between classes I (endo-class) and II (PM-class), as shown in **Figure 1**.

GO enrichment analysis was conducted to uncover the potential functions of the *NHX* genes. Among the twenty *TaNHX* genes, significant enrichment was observed in biological processes (BP), molecular functions (MF), and cellular components (CC). In BP, the enriched terms included “sodium ion transmembrane transport,” “response to salt stress,” “potassium ion homeostasis,” and “regulation of pH,” among others (**Supplementary Table S3**). The enriched MF terms encompassed “binding,” “antiporter activity,” and “potassium: proton antiporter activity.” Furthermore, the enriched CC terms were associated with “plasma membrane” and “vacuolar membrane.” Notably, individual *TaNHX* genes exhibited enrichment in specific GO terms, indicating their involvement in distinct processes. For comprehensive information on the enriched GO terms and their functions see **Supplementary Table S3**.

### 3.2 Analysis of conserved motifs, gene structure, cis-regulatory elements, and physical mapping

**Figure 2:**
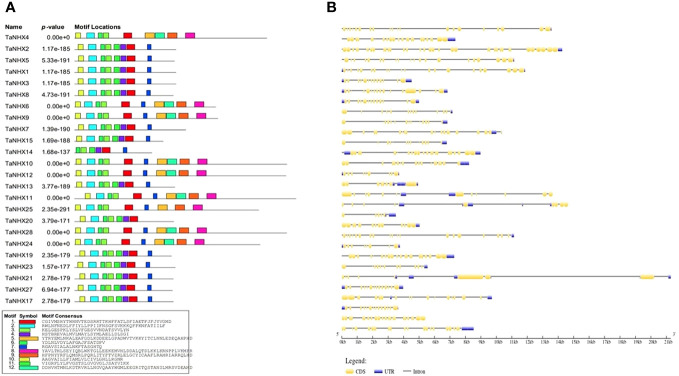
**(A)** Distribution of conserved motifs identified in proteins encoded by TaNHX. **(B)** Gene structures showing the organization of exons and introns, *TaNHX* genes.

The MEME web-server with default settings was employed to predict conserved motifs, shedding light on the evolutionary preservation of specific functional amino acids within the NHX protein family. Based on the protein sequences of all NHXs, we discovered a total of 10 potential motifs (**Figure 2A**). The length of the TaNHXs’ anticipated motifs ranged from 6 to 50 amino acids. Members of the same subfamily shared a similar motif arrangement. Logos of the motifs were presented in **Supplementary Figure S1**. The descriptive details of domains were given in **Supplementary Table S4**.

Using Gene Structure Display Server v.2.0, the intron/exon architecture of the genes was examined to determine the structural properties of the wheat *TaNHX*s. The number of introns and exons differed significantly depending on the intron/exon patterns studied, which further explains the diversity in gene length. Non-coding sequences are frequently found in the genome, which is thought to be a sign of genomic complexity. Thus, examining these intron configurations reveals important details about the development, regulation and functionality of the NHXs. The examination of the wheat *TaNHX* gene architectures revealed significant variations across the three classes in terms of the number of introns and exons. The bulk of the Vac-class family among the 30 wheat *TaNHX*s included UTR (untranslated region) sequences at both the 5’ and 3’ ends. However, 6 out of the 12 Vac-class *TaNHX*s had 14 exons, 3 had more than 20 exons, and the rest were less than 10. The endo-class *TaNHX* had more than 10 exons except *TaNHX26* (**Figure 2B**). However, the PM-class *TaNHX* (*TaNHX9* and *TaNHX10*) had the highest share of intron-exons, with 27, 37 exons and 14, 20 introns, respectively. It was found that the distribution of introns and exons in the genes belonging to the same clade was very comparable. The exon lengths and intron regions of genes in the same class were mostly conserved. The analysis of amino acid sequence identity further confirmed the wheat TaNHXs’ sequence conservation (**Figure 2B**).

**Figure 3:**
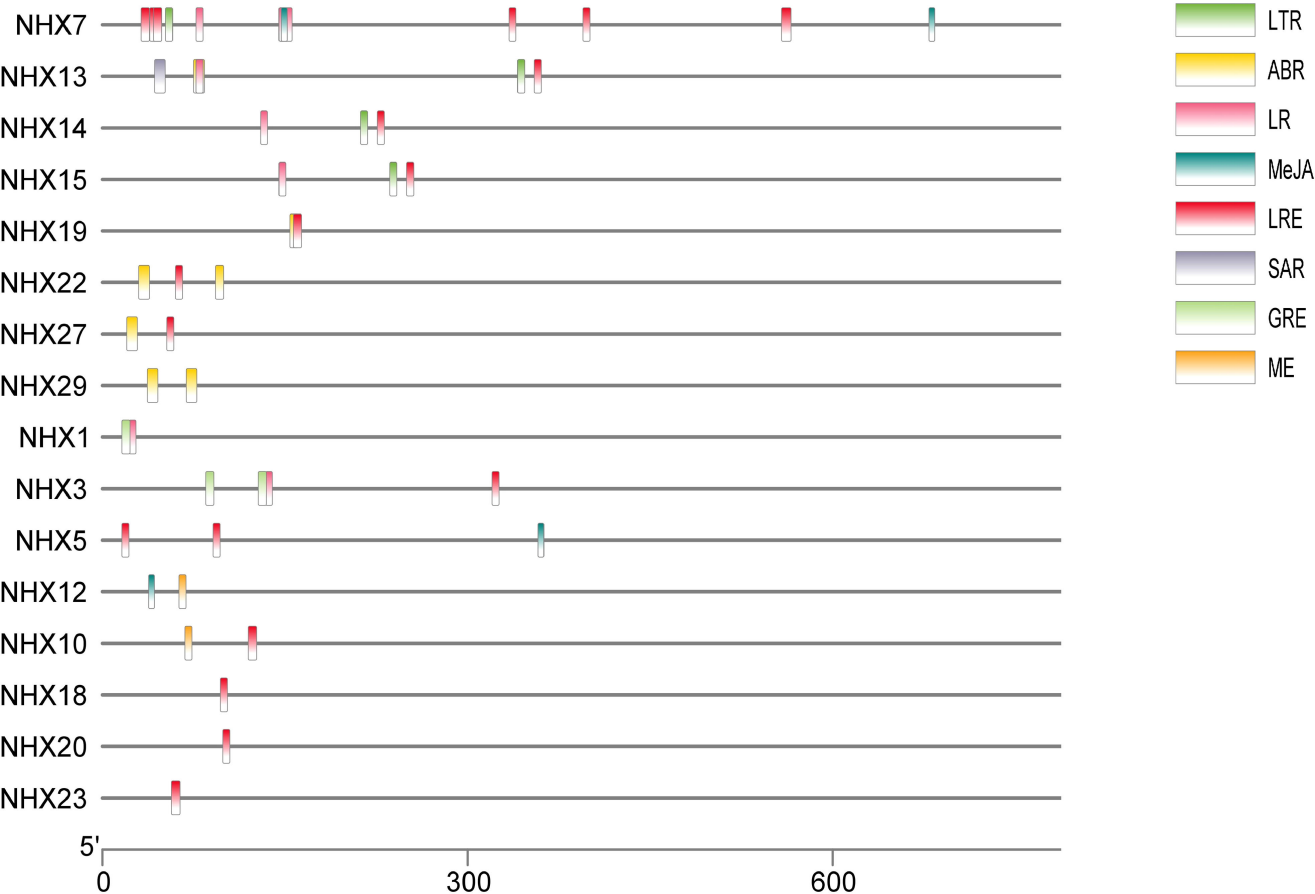
Predicted *cis*-elements in TaNHX promoters. The upstream length to the translation starting site can be inferred according to the scale at the bottom. The green, yellow, pink, red, orange and violet-colored boxes stand for DSR, SA, MeJA, Me, ABRE and MYB cis-elements, respectively.

In order to better understand transcriptional control and gene expression, the promoter sequences of wheat *TaNHX* were identified by PlantCARE software (**Figure 3**). The major focus of the analysis was on *cis*-acting components associated with stress and hormones. Twenty two of the *cis*-acting components that were discovered had a hormonal connection, including ABA, salicylic acid (SA), gibberellin (GA), and jasmonate. The *TaNHX* comprised of phytohormones were *TaNHX1, TaNHX3, TaNHX5, TaNHX7, TaNHX12, TaNHX13, TaNHX19, TaNHX22, TaNHX23*, and *TaNHX29* (**Figure 3**). Eleven *cis*-acting components, including anaerobic environments and low temperatures, were responsible for stress. The number of light-responsive elements was higher among these components. These cis-acting elements’ findings imply that *TaNHX*s may be crucial for the control of hormones and the response to stress in wheat.

**Figure 4:**
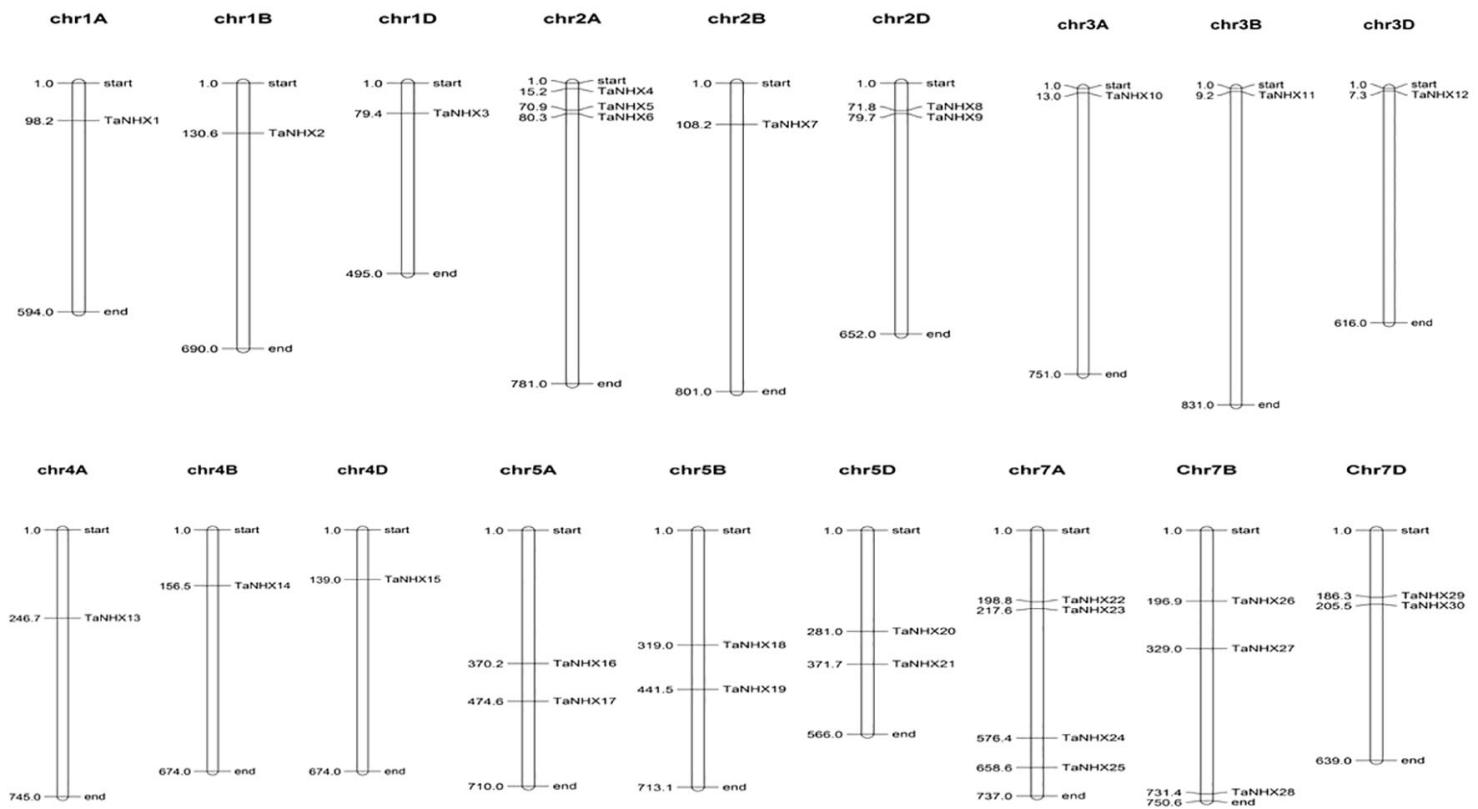
The distribution of NHX genes on chromosomes of *T. aestivum*. Chromosome numbers have been indicated on the top of each chromosome. The position of each gene on the respective chromosome has been depicted in terms of mega base-pairs by numbers beside each gene.

To better comprehend how the *TaNHX* gene family evolved, we further examined the gene duplication occurrences. Thirty *TaNHX* genes were unevenly mapped onto six (A, B, D) chromosomes of the 21 *T. aestivum* chromosomes (**Figure 4**). Two chromosomes (Chr2 and Chr5) contained six *TaNHX* genes and three chromosomes (Chr1, Chr3, and Chr4) contained only one *TaNHX* gene on each A, B, and D sub-genome (**Figure 4**). No genes were present on chromosome 6. Gene duplication events, which are a primary mechanism of gene family growth, gave chances for the synthesis of additional genes and their functional divergence.

### 3.3 Protein-protein interaction, miRNA targeting, and duplication events in NHX genes

**Figure 5:**
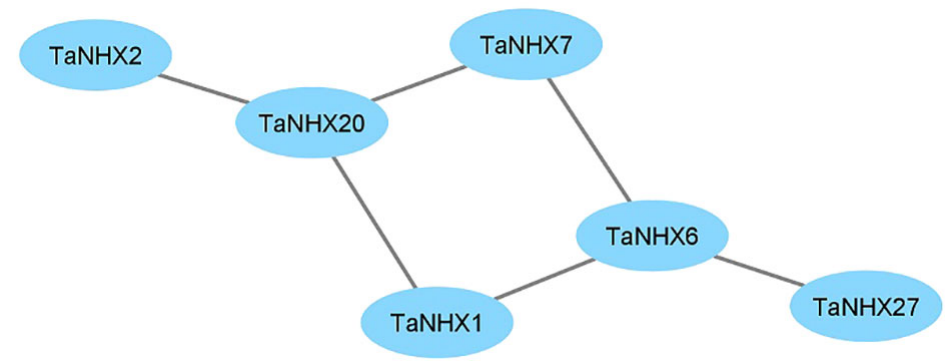
Interaction network among NHX protein in wheat.

The response to salt stress is one of the major biological processes in which NHXs play a significant role. The PPI network was created by the STRING database to further examine the potential role of TaNHXs during potential interactions with other proteins (**Figure 5**). TaNHX proteins are not expected to have any direct interactions with one another. Out of thirty TaNHX proteins, six were participating in the protein-protein interaction network. They shared a similar kind of putative interactive protein. TaNHX1, TaNHX2, TaNHX7, and TaNHX20 were involved in proton exchange and belong to monovalent and proton antiporters (**Figure 5**). Numerous physiological processes, including vesicle trafficking, pH control, K^+^ homeostasis, protein transport, and growth and development, are regulated by the antiporters. In contrast, TaNHX6 interacted with SOS1, which is crucial for the expulsion of Na^+^ ions from cells.

This study employed network analysis, using *TaNHX* gene expression data, to uncover key insights into sodium ion transport mechanisms in plants. Significant Gene Ontology (GO) terms (FDR < 0.001) were identified, revealing *TaNHX* genes’ involvement in cellular processes, particularly transmembrane transport. Intriguingly, our analysis revealed intricate interactions between sodium ion transport and the regulation of biological quality, chemical homeostasis, and metal ion homeostasis (**Supplementary Figure S2**). Subcellular localization indicated that *TaNHX* proteins primarily reside in plasma membranes, endosomes, and vacuoles, emphasizing their role in Na^+^/-H antiporter activity and ion homeostasis. The findings also imply potential gene interactions affecting activity levels, paving the way for further investigations into these regulatory networks in plant sodium ion transport (**Supplementary Figure S2**). This research contributes valuable insights to plant biology and ion homeostasis regulation.

The psRNATarget service has been used to look at how miRNAs regulate the expression of *TaNHX* genes. For twenty distinct miRNAs, we identified 32 *TaNHX* genes as potential targets (**Table 2**). Tae-miR395 is implicated in the regulation of eight *TaNHX* genes (*TaNHX6, TaNHX9, TaNHX10, TaNHX11, TaNHX12, TaNHX24, TaNHX25*, and *TaNHX28*). Tae-miR9666 accounted for regulating the expression of four *TaNHX* genes (T*aNHX6, TaNHX9, TaNHX24*, and *TaNHX25*). The expression of *TaNHX* genes (*TaNHX1, TaNHX3*, and *TaNHX29*) may be influenced by Tae-miR9656 (**Table 2**). Tae-miR9772, Tae-miR9773, and Tae-miR9774 were predicted to regulate the expression of *TaNHX22*, *TaNHX13*, and *TaNHX9*, respectively.

**Table 2:** Prediction of Tae-MIR genes and their targets by using the psRNATarget server with default parameters.

miRNA_Acc.	Target_Acc.	Target_start	Target_end	miRNA_aligned_fragment	Target_aligned_fragment	Inhibition
tae-miR9773	TaNHX13	2688	2710	UUUGUUUUUAUGUUAUUUUGUGA	AAACCAGAAACCAUAAAAACAAA	Cleavage
	TaNHX13	2334	2356	UUUGUUUUUAUGUUAUUUUGUGA	UUUGAAAAUGACGUAAUAAUAAU	Cleavage
tae-miR395a	TaNHX24	1731	1751	GUGAAGUGUUUGGGGGAACUC	AAGUUUUAUCACAUACUUCAC	Translation
	TaNHX25	1674	1694	GUGAAGUGUUUGGGGGAACUC	AAGUUUUAUCACAUACUUCAC	Translation
	TaNHX9	1731	1751	GUGAAGUGUUUGGGGGAACUC	AAGUUUUAUCACAUACUUCAC	Translation
	TaNHX6	1731	1751	GUGAAGUGUUUGGGGGAACUC	AAGUUUUAUCACAUACUUCAC	Translation
	TaNHX24	549	569	GUGAAGUGUUUGGGGGAACUC	AAGUUUUAUCACAUACUUCAC	Translation
	TaNHX11	2244	2264	GUGAAGUGUUUGGGGGAACUC	ACAUUCCCUCAGGUGCUUCGU	Cleavage
	TaNHX11	2226	2246	GUGAAGUGUUUGGGGGAACUC	ACAUUCCCUCAGGUGCUUCGU	Cleavage
	TaNHX10	2095	2115	GUGAAGUGUUUGGGGGAACUC	ACAUUCCCUCAGGUGCUUCGU	Cleavage
	TaNHX10	1969	1989	GUGAAGUGUUUGGGGGAACUC	ACAUUCCCUCAGGUGCUUCGU	Cleavage
	TaNHX12	2080	2100	GUGAAGUGUUUGGGGGAACUC	ACAUUCCCUCAGGUGCUUCGU	Cleavage
	TaNHX12	2065	2085	GUGAAGUGUUUGGGGGAACUC	ACAUUCCCUCAGGUGCUUCGU	Cleavage
	TaNHX28	2031	2051	GUGAAGUGUUUGGGGGAACUC	ACAUUCCCUCAGGUGCUUCGU	Cleavage
tae-miR9656-3p	TaNHX1	1822	1842	CUUCGAGACUCUGAACAGCGG	ACGCUGUGUAGAGUUUUGGGA	Cleavage
	TaNHX3	2166	2186	CUUCGAGACUCUGAACAGCGG	ACGCUGUGUAGAGUUUUGGGA	Cleavage
	TaNHX29	1745	1765	UUUAUGAUCACUCUCGUUUUG	GGAGACCAGAGUGCUUAUAAU	Cleavage
tae-miR395b	TaNHX11	2244	2263	UGAAGUGUUUGGGGGAACUC	ACAUUCCCUCAGGUGCUUCG	Cleavage
	TaNHX11	2226	2245	UGAAGUGUUUGGGGGAACUC	ACAUUCCCUCAGGUGCUUCG	Cleavage
	TaNHX10	2095	2114	UGAAGUGUUUGGGGGAACUC	ACAUUCCCUCAGGUGCUUCG	Cleavage
	TaNHX10	1969	1988	UGAAGUGUUUGGGGGAACUC	ACAUUCCCUCAGGUGCUUCG	Cleavage
	TaNHX12	2080	2099	UGAAGUGUUUGGGGGAACUC	ACAUUCCCUCAGGUGCUUCG	Cleavage
	TaNHX12	2065	2084	UGAAGUGUUUGGGGGAACUC	ACAUUCCCUCAGGUGCUUCG	Cleavage
	TaNHX28	2031	2050	UGAAGUGUUUGGGGGAACUC	ACAUUCCCUCAGGUGCUUCG	Cleavage
	TaNHX24	1731	1750	UGAAGUGUUUGGGGGAACUC	AAGUUUUAUCACAUACUUCA	Cleavage
	TaNHX25	1674	1693	UGAAGUGUUUGGGGGAACUC	AAGUUUUAUCACAUACUUCA	Cleavage
	TaNHX9	1731	1750	UGAAGUGUUUGGGGGAACUC	AAGUUUUAUCACAUACUUCA	Cleavage
	TaNHX6	1731	1750	UGAAGUGUUUGGGGGAACUC	AAGUUUUAUCACAUACUUCA	Cleavage
	TaNHX24	549	568	UGAAGUGUUUGGGGGAACUC	AAGUUUUAUCACAUACUUCA	Cleavage
tae-miR7757-5p	TaNHX30	881	902	AUAAAACCUUCAGCUAUCCAUC	ACAUGCUAGCUGAAGGCUUUGG	Cleavage
	TaNHX26	800	821	AUAAAACCUUCAGCUAUCCAUC	ACAUGCUAGCUGAAGGCUUUGG	Cleavage
	TaNHX26	422	443	AUAAAACCUUCAGCUAUCCAUC	ACAUGCUAGCUGAAGGCUUUGG	Cleavage
tae-miR9666b-3p	TaNHX24	1154	1176	CGGUUGGGCUGUAUGA-UGGCGA	GUGCUAGUGAUACAGCUCAACCU	Cleavage
	TaNHX25	1175	1197	CGGUUGGGCUGUAUGA-UGGCGA	GUGCUAGUGAUACAGCUCAACCU	Cleavage
	TaNHX9	1154	1176	CGGUUGGGCUGUAUGA-UGGCGA	GUGCUAGUGAUACAGCUCAACCU	Cleavage
	TaNHX6	1154	1176	CGGUUGGGCUGUAUGA-UGGCGA	AUGCUAGUGAUACAGCUCAACCU	Cleavage
tae-miR9772	TaNHX22	481	501	UGAGAUGAGAUUACCCCAUAC	GUAUUCGGUGUUUUCAUCUCG	Translation
tae-miR9773	TaNHX13	2299	2321	UUUGUUUUUAUGUUAUUUUGUGA	UCGGUAUAUACCAUAAAAACCAA	Cleavage
tae-miR9774	TaNHX9	1584	1605	CAAGAUAUUGGGUAUUUCUGUC	UCAAGCUACAGCCAAUAUUUUG	Cleavage

**Figure 6:**
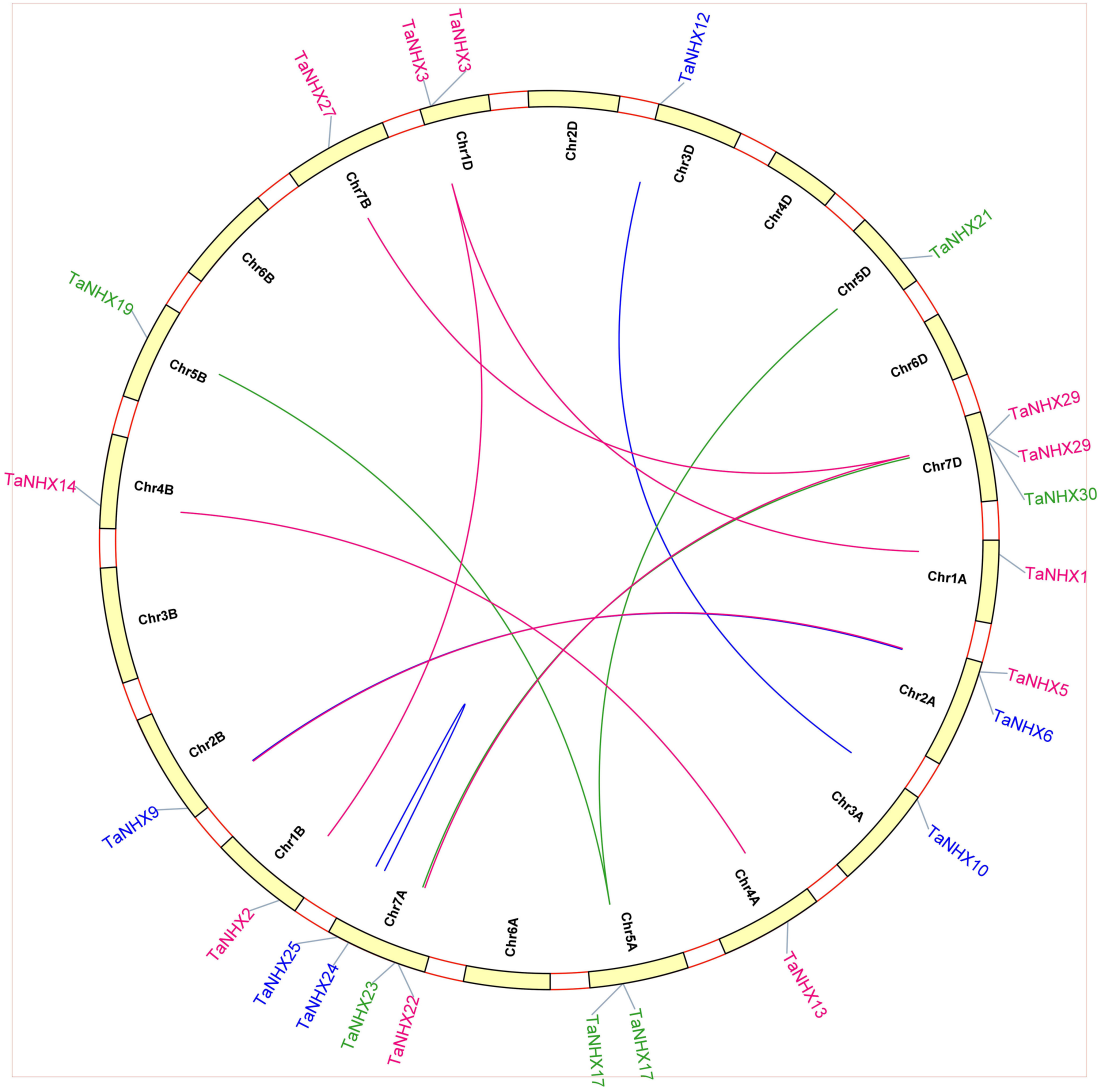
Interspecies synteny of *T. aestivum*, *A. thaliana*, *O. indica*, and *H. vulgare* on the basis of orthologous genes. Gray lines in the background indicate the collinear blocks within *T. aestivum*, *A. thaliana*, *O. sativa, and H. vulgare* while the red lines highlight the syntenic *NHX* gene pairs.

Comparative analysis was utilized to evaluate the orthologous of *TaNHX* gene duplication in wheat and *A. tauschii*. We discovered 28 orthologous gene pairs among all the *NHX* genes from wheat and *A. tauschii*. Contrarily, there were 27 *NHX* orthologous gene pairs discovered between wheat and rice, 29 between wheat and *T. dicoccoides*, and 30 between wheat and Arabidopsis (**Supplementary Table S4**). Orthologous associations between several species can be found through the analysis of collinearity correlations. *NHX* gene pairings between the genomes of *T. aestivum* and *A. thaliana* were syntenized. The findings revealed that 12 *T. aestivum NHX* genes shared syntenic relationships with *AtNHX* genes (**Figure 6**), indicating that these genes may have aided in the development of the *TaNHX* gene family.

**Figure 7:**
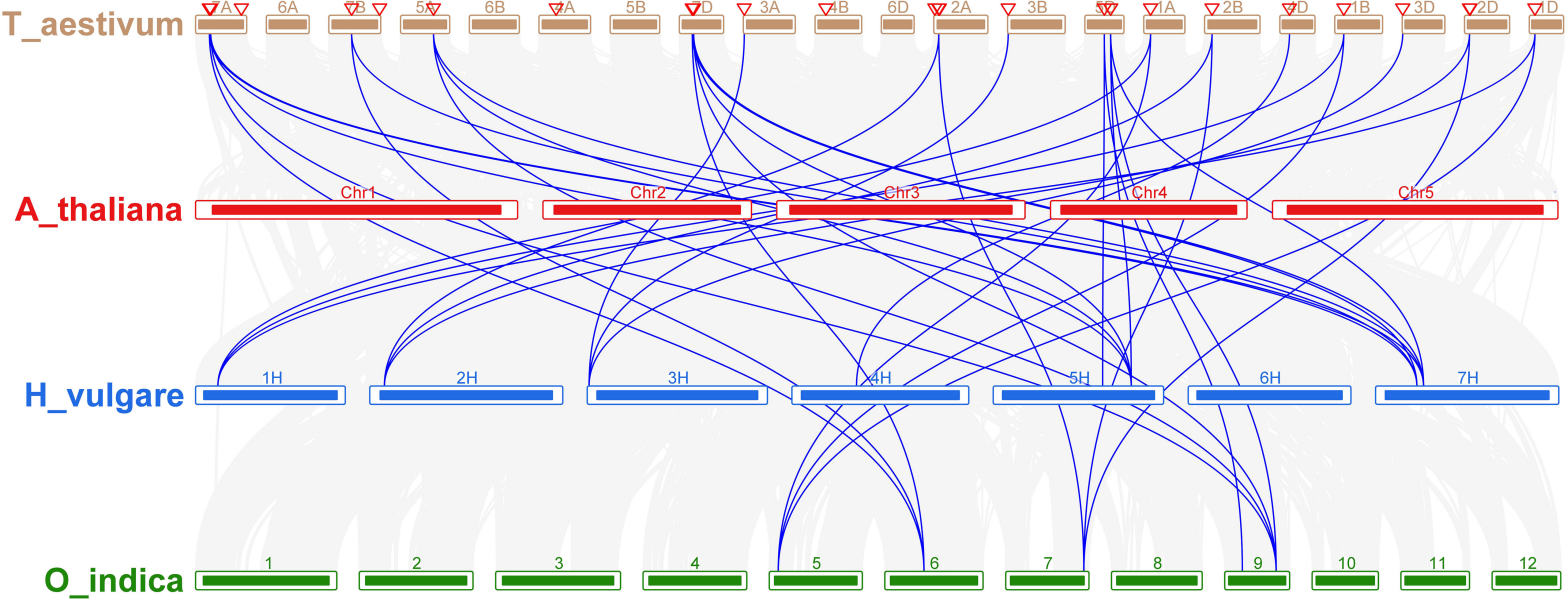
The synteny analysis of TaNHX family in *T. aestivum*. Different colors represent NHX family on A, B and D sub-genome red lines indicate duplicated *TaNHX* gene pairs on A sub-genome, green lines indicated on B sub-genome and blue represented D sub-genome. The chromosome number is indicated at the bottom of each chromosome.

We conducted an analysis of synonymous substitutions (K_s_) and non-synonymous substitutions (K_a_) values to investigate the selective pressures underlying the duplication events of *TaNHX* genes, considering all nucleotide sequences. Our findings unveiled intriguing patterns, notably identifying six gene pairs (*TaNHX7/TaNHX5, TaNHX15/TaNHX14, TaNHX22/TaNHX27, TaNHX1/TaNHX3, TaNHX20/TaNHX18*, and *TaNHX26/TaNHX23*) characterized by K_a_/K_s_ ratios lower than 1. These results suggest that these gene pairs experienced purifying selection, indicative of their evolutionary conservation (**Supplementary Table 5**). Our analysis detected nine distinct segmental duplication events occurring across various chromosomes, along with one notable tandem duplication event within the same chromosome. These findings underscore the pivotal role of segmental duplications in driving the expansion of *TaNHX* genes within the wheat genome. Furthermore, our observations suggest that certain *TaNHX* genes may have originated as a consequence of gene duplication events, shedding light on the evolutionary mechanisms responsible for the diversification of this gene family. But it may be due to selection pressure that the number of *TaNHX* genes was not expended in comparison to other transcription factors in wheat. Genes from three subfamilies (PM, endo and vac.) participated in the tandem and segmental duplication. We also investigated how frequently tandem duplications occur. This region contained 10 *TaNHX* gene pairs, all of which were closely linked. Notably, the sequence identities among these genes exceeded 80%, strongly suggesting their involvement in tandem duplication events. Given the profound impact of gene duplication on the emergence of novel functionalities and gene families, we conducted a comprehensive exploration of *TaNHX* gene duplication events within the wheat genome. The paralogous gene pairs were employed to construct a circos plot (**Figure 7**), enabling us to visualize and elucidate the intricate patterns of duplication events and their implications.

### 3.4 Tissue-specific expression profiles of NHX genes

**Figure 8:**
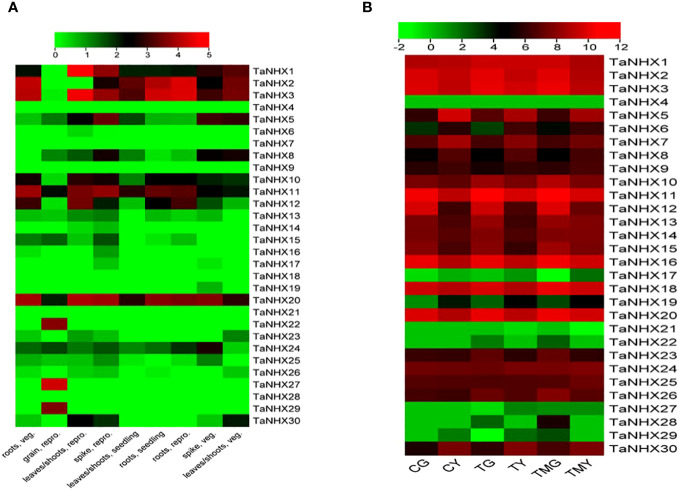
Normalized expression profiles of *NHX* genes of *T. aestivum* in various plant tissues under; **(A)** control and **(B)** salt stress conditions. CG represented the control wheat root samples, TG: 150mM NaCl treated wheat root, TMG: 5mM 3-Methylamide + 150mM NaCl treated wheat root, CY: control wheat leaves, TY: 150mM NaCl treated wheat leaves, TMY: 5mM 3-MA + 150mM NaCl treated leaves.

*TaNHX* expression levels were analyzed in wheat roots and leaf tissues exposed to salt stress. The expression analysis was done using RNA-seq data (SRP304900) from NCBI. Under normal conditions, *TaNHX1, TaNHX2, TaNHX3, TaNHX5, TaNHX10, TaNHX11*, and *TaNHX20* were found to be upregulated in all tissues except in grain (**Figure 8**). However, *TaNHXs* displayed differential gene expression levels among the tissue types in different genes. It was observed that 23 out of 30 *TaNHX* genes were expressed very highly in both tissues. Whereas, the genes e.g., *TaNHX4, TaNHX17, TaNHX21, TaNHX22, TaNHX27, TaNHX28*, and *TaNHX29* expression were downregulated in all tissues under salt stress conditions (**Figure 8**). However, it was found that *TaNHX3, TaNHX5, TaNHX7, TaNHX11, TaNHX16, TaNHX18*, and *TaNHX20* expressed upregulation in all tissues under heat, drought, and cold (**Supplementary Figure S3**) stresses.

### 3.5 Validation of NHX genes using qRT- PCR under salinity stress

**Figure 9:**
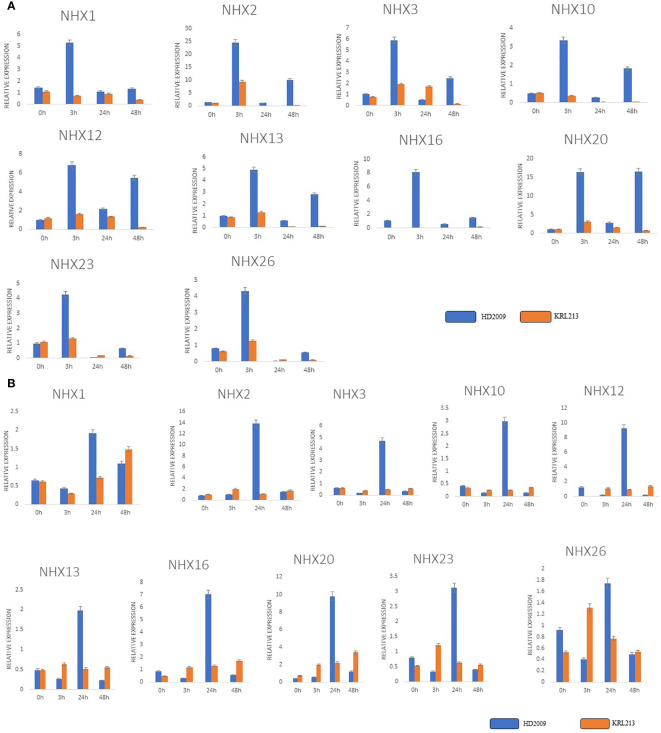
qRT-PCR expression analysis of selected genes in contrasting wheat varieties KRL213 and HD2009 for salt tolerance. **(A)** leaf tissues and, **(B)** root tissues under salt stress (150mM NaCl) at different time intervals. The names of the genes are shown in the x-axis, and y-axis represents the fold changes of expression of the genes. The data represents the mean ± standard deviation with n = 3. * Above the bars indicate significant correlation at the 0.05, as analyzed by Student’s T-test.

Using qRT-PCR analysis, we quantitatively evaluated the expression patterns of ten *TaNHXs* genes under control and salt-stress conditions to observe the potential functions that TaNHXs may play in wheat in responses to salinity stress. The findings demonstrated that salt tolerance (KRL213) and salt-sensitive (HD2009) wheat cultivars both stimulated all *TaNHX* genes in leaf and root tissues in a concentration-dependent manner (**Figure 9**). The tissues with the highest induction were the roots, followed by the leaves. *TaNHX* gene overexpression caused by salinity was greater in KRL213 than HD2009. Out of ten *TaNHX* genes, five genes [*TaNHX2* (~14 folds), *TaNHX12* (~9 folds), *TaNHX16* (~7 folds), *TaNHX20* (~10 folds), *TaNHX23* (~3 folds)] showed up-regulation, in the roots of KRL213 at 48 h under 150 mM NaCl stress condition (**Figure 9A**). On the other hand, expression levels of *TaNHX2, TaNHX16*, and *TaNHX20* were expressed more than twofold in roots treated with 150 mM NaCl at different time intervals. It was observed that *TaNHX2, TaNHX12*, and *TaNHX20* expression levels were higher in the leaves of KRL213 (~10, ~5, and ~16 times, respectively) than HD2009 were down-regulated at 48 h of 150 mM NaCl treatment (**Figure 9B**). Salinity stress clearly shows up-regulation of TaNHXs in the leaves of KRL213, whereas in HD2009 two genes *TaNHX2* and *TaNHX20*, were expressed higher in leaf tissue at 0 h and 3 h of stress. In KRL213 leaf tissue, all genes used for the validation were up-regulated at 3 h of salt stress (**Figure 9B**). The gene expression profiles of individual *TaNHX* members were evaluated in both leaf and root tissues under salinity stress conditions, yielding results that consistently mirrored the salt tolerance characteristics of the two wheat genotypes under study.

### 3.6 MD simulation analysis

**Figure 10:**
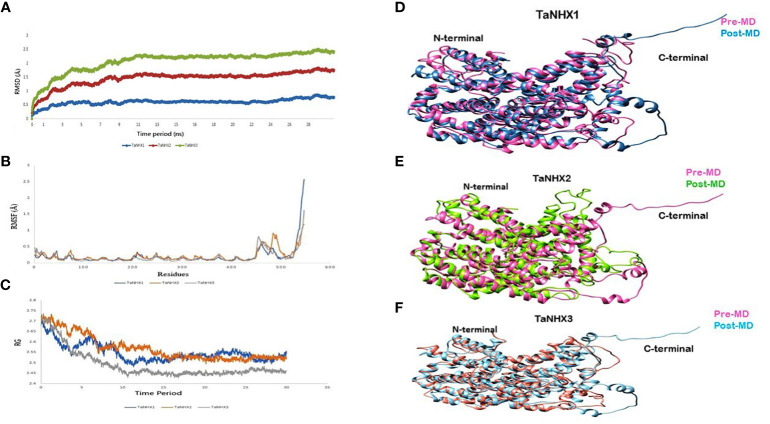
MD simulations analysis of NHX Proteins. **(A)** Root mean square deviation, **(B)** Root mean square fluctuations **(C)** Radius of gyration during 30 ns simulations. Superimposition between pre- and post-MD structure shown with different colours for; **(D)** TaNHX1, **(E)** TaNHX2 and, **(F)** TaNHX3 proteins.

The Root Mean Square Deviation (RMSD) serves a crucial statistic for assessing the average changes of a set of atoms concerning a reference frame. Analyzing the RMSD of the protein backbone atoms relative to their initial positions can yield valuable insights into the protein conformational dynamics. In our investigation, the RMSD values for all three proteins were below 2.5 nm. **Figure 10A** illustrates a stabilizing trend in the RMSD values around 10 ns mark, suggesting the attainment of equilibrium within the simulation. This stabilization milestone serves as an optimal foundation for subsequent analysis.

Moreover, the Root Mean Square Fluctuation (RMSF) analysis delves into the localized fluctuations occurring along the protein chain during the molecular dynamics (MD) simulations RMSF graph uncovered fluctuations primarily at the C-terminals of the proteins, while the core of the TaNHX proteins displayed no substantial variations (**Figure 10B**). Additionally, the radius of gyration (Rg) value for the TaNHX structure exhibited a decreasing trend as the simulation time progressed, indicating a progressive compaction of the modeled structure. This observation aligns with the results from the initial run of the simulations. The radius of gyration is a measure of the overall size and compactness of a protein structure. A lower Rg value suggests a more tightly folded and compact conformation, while a higher Rg value indicates a more extended or flexible structure. Therefore, the lower Rg values obtained for the TaNHX protein structure suggest that they adopt a more compact and stable conformation (**Figure 10C**).

## 4 Discussion

Sodium-proton (Na^+^/H^+^) antiporters, which are encoded by genes belonging to the NHX family, play a pivotal role in upholding ion equilibrium and aiding in membrane trafficking. Their significance is particularly pronounced in plant cells exposed to the challenges of salinity stress, as elucidated by Pehlivan et al. (2016). The *NHX* gene families have undergone comprehensive identification and functional characterization in various plant species such as Arabidopsis, rice, wheat, sugar beet, and cotton (Manik et al., 2015; Wilkins et al., 2016; Kumari et al., 2018; and Wu et al., 2019). A comprehensive genome-wide investigation of *TaNHX* genes in *T. aestivum* has not been conducted yet. Our study aimed to fill this gap by identifying *TaNHX* genes in the wheat genome and conducting a thorough characterization, including phylogenetic relationships, their characterization, and expression profiling under varying salinity stress conditions, along with molecular dynamic simulation.

Our analysis revealed that *TaNHX* members in wheat could be classified into three groups based on multiple sequence alignments and sub-cellular localization. For instance, *TaNHX7* and *TaNHX8* were found to be localized in the plasma membrane, *TaNHX5* and *TaNHX6* in the endo-membrane, while the remaining members were localized in the vacuole. This localization pattern was comparable with the prior studies in Arabidopsis (Ramesh et al., 2019; Parveen et al., 2021). Notably, the similarity observed in NHX families across lower to higher plant taxa suggests the retention of functional importance throughout evolutionary processes (Shuai et al., 2013). Sub-cellular localization of NHX transporters significantly influences their functionality. NHX family members situated on both the plasma membrane and tonoplast play an integral role in maintaining ionic equilibrium by sequestering and eliminating excessive sodium ions (Na^+^).

In our analysis, we identified ten conserved motifs in TaNHXs. Notably, a key element of profound importance is the remarkably conserved membrane-spanning pore and cation-binding domain, exemplified by the nonapeptide sequence “FFIYLLPPI,” renowned as the amiloride-binding site. This distinctive characteristic defines the membrane-bound NHX transporters in plants (Wu et al., 2011). This site is well-known for its role in inhibiting cation/H^+^ exchange when amiloride is present. Interestingly, we found that this region is conserved in the second motif of all TaNHX peptides, indicating that TaNHXs, as a whole, exhibit functional and sequence-based similarities, with the exception of *TaNHX14* (**Figure 2A**). Similar sequence characteristics have been observed in Arabidopsis (Aharon et al., 2003), soybean (Chen et al., 2015), and tea (Paul et al., 2021). These findings contribute to our understanding of the conserved motifs and functional characteristics of TaNHX proteins in wheat and highlight the evolutionary relationships among TaNHXs based on their gene structures.

*cis*-acting elements, serving as genetic switches for gene transcription, exert significant control over biological processes, encompassing responses to hormonal fluctuations, environmental stress, and developmental stresses (Ren et al., 2017; Zhang et al., 2020). Phytohormones such as gibberellins, methyl jasmonic acid, and abscisic acid wield immense influence over growth, development, and stress responses (Zhao et al., 2021). It is conceivable that TaNHXs actively participate in hormone signaling throughout wheat’s developmental stages and stress responses. This conjecture finds support in the presence of ABREs (abscisic acid responsive elements) and MeJA (methyl jasmonate) responsive elements within the promoter regions of these genes (**Table 2**). Our findings are in tune with those in *P. trichocarpa* (Shuai et al., 2013) and *S. bicolor* (Kumari et al., 2018), which unveiled stress-responsive elements in *NHX* gene promoters. Furthermore, the promoter regions of *TaNHX*s harbor elements associated with light responsiveness and low-temperature sensitivity, suggesting their potential involvement in pivotal regulatory processes encompassing phytohormonal dynamics, stress responses, cellular development, and metabolism. By identifying these *cis*-acting elements and their association with TaNHXs in wheat, our study sheds light on their potential role in the regulation of *TaNHX* gene expression and their involvement in crucial physiological processes. These findings deepen our understanding of the intricate signaling networks that coordinate hormone responses, stress adaptation, and overall plant development in wheat.

The association of TaNHXs with other proteins, such as SOS2, contributes to the production of a protein kinase that plays a vital role in alleviating salt stress in Arabidopsis (Wilkins et al., 2016). Another protein, SOS3, acts as a calcium ion sensor and facilitates the transportation of SOS2 and SOS1, leading to the efflux of sodium ions from the cells (Shi et al., 2000). The analysis of protein-protein interactions (PPI) revealed a predicted interaction between PM-bounded TaNHX6 and SOS1. TaNHX6, known to possess several hormone- and stress-related *cis*-acting elements, plays a pivotal role in facilitating the exclusion of Na^+^ ions from the cell. Similar results have been observed by Manik et al. (2015) in honeysuckle. However, it is important to note that the regulation of salt tolerance involves a complex and interconnected network of interactions (**Figure 5**). Among the key players in this network are the membrane-bound pyrophosphatases, namely H^+^-PPase and H^+^-ATPase related proteins. These proton transporters, dependent on potassium, are crucial for plant responses to stress as well as growth and development. A balanced Na^+^/K^+^ ratio is essential for various vital functions, including proper stomatal function, protein synthesis, cell osmoregulation, photosynthesis, and turgor maintenance (Parveen et al., 2021). Given the importance of TaNHXs and potassium transport-related proteins in maintaining this balance, it is justified to include them in protein interaction networks in current research endeavors.

miRNAs have been previously reported as having abiotic stress-responsiveness (Ramesh et al., 2019), indicating their potential involvement in regulating stress-related processes. Specific miRNA families have known roles in different aspects of plant development and response to abiotic stresses (Shuai et al., 2013; Gupta et al., 2014; Luan et al., 2014; Zhang and Chen, 2021; Liu et al., 2022). We identified a total of 20 different miRNAs targeting 26 distinct *TaNHX* genes (**Table 2**). Differential expression patterns of *TaNHX* genes observed in our study can be linked to the stress-responsive miRNAs that target these genes, further emphasizing their role in stress adaptation and plant development.

The analysis of 30 salt-responsive genes in *T. aestivum* revealed interesting insights. Collinearity analysis indicated that 18 *TaNHX* genes resulted from whole-genome duplication or segmental duplication, a pattern observed in various gene families, including cotton MADS-Box, GT47, and soybean WRKY, highlighting the role of gene duplication in their evolution (Cannon et al., 2004; Yin et al., 2013; Ren et al., 2017; Wu et al., 2019). Additionally, the collinearity analysis of NHXs revealed shared ancestry among the A, B, and D genomes of NHXs, signifying their common origin. These findings shed light on the NHX family’s evolution in wheat, primarily driven by WGD or segmental duplication events. This deepens our understanding of gene duplication mechanisms, genetic information exchange between species, and the processes shaping species evolution. Furthermore, the evaluation of non-synonymous (K_a_) and synonymous (K_s_) substitution rates among duplicated gene pairs stands as a pivotal tool for assessing selection pressure and approximating the timing of duplications. In the context of *TaNHX* genes in wheat, these findings robustly suggest that purifying selection mechanisms played a prominent role in generating genes with conserved functions and, in some cases, undergoing pseudogenization (**Supplementary Table S4**). Regarding the anticipated motifs within NHX proteins, it has become evident that genes within the duplicated gene cluster showcase functional conservation, while a few motifs lack discernible functional details, potentially indicating pseudogenization. This intriguing observation underscores the possibility of pseudogenization events, as indicated in (**Supplementary Table S4**). This phenomenon can be attributed to one or more ancestral polyploidy events that transpired in numerous angiosperm plant lineages. Consequently, these gene duplications within the wheat genome have been instrumental in the emergence of evolutionary innovations. Furthermore, a thorough examination of syntenic blocks among NHX genes across wheat and several other plant species has unveiled that the closest orthologs of the wheat channels trace their origins to barley. These extensive synteny relationships at the gene level serve as compelling evidence affirming the close evolutionary affiliations between these species (Ullah et al., 2022). Variations in these evolutionary relationships can provide valuable insights into the substantial rearrangement events that have left their imprint on the genomes of wheat and its related species throughout the course of evolution.

Considering the association of *TaNHX* genes with stress-responsive *cis*-acting elements and miRNAs, we hypothesized that their expression patterns would exhibit variations. In our study, we observed up-regulation of *TaNHX* genes in both leaf and root tissues of the cultivars under salinity stress, irrespective of the severity of salt exposure and the cultivars’ salt tolerance capacity (**Figure 8A**). Remarkably, the roots, being the first organs exposed to salt, displayed significantly higher induction of *TaNHX* genes. Although the induction of TaNHXs in leaves was relatively lower compared to roots, it still contributed to the exclusion and improved compartmentalization of Na^+^ ions within the cells. This heightened expression of sodium transporters directly facilitated the maintenance of Na^+^ ion homeostasis. Additionally, we observed a gradual increase in gene expression within 3h of salt treatment in leaf tissue. Similar expression patterns were observed in cotton roots, where gene expression reached its peak at 3h, gradually declined, and then increased again at 48h (**Figure 8**). These findings align with previous studies on *BvNHX* genes in sugar beet (Wu et al., 2019). Similarly, the wheat gene *TaNHX3* exhibited increased expression within 24h in both roots and leaves, followed by a gradual decrease within 48h. Consistent with previous studies on wheat transgene TaNHX, our findings highlighted the responsiveness of *TaNHX2, TaNHX12*, and *TaNHX20* to salinity stress. Yue et al. (2021) found that the addition of 3-methyladenine (3-MA) inhibits autophagy, increases ROS accumulation and impairs the tolerance of wheat seedlings to NaCl stress. Differential expression patterns of NHXs under salinity stress have also been reported in other plant species, such as *Beta vulgaris* with five *NHX*s (Wu et al., 2019), *Populus trichocarpa* with eight *NHXs* (Tian et al., 2017), and *Sorghum bicolor* with six *NHXs* (Kumari et al., 2018). Hierarchical clustering analysis (**Figure 8B**) revealed two distinct clusters based on the activities of TaNHXs in all tissues under saline conditions in both cultivars. This clustering suggests different regulatory mechanisms or functional roles of TaNHXs in response to salinity stress.

To gain further insights into the TaNHX proteins, we employed molecular modeling and MD simulations to analyze their three-dimensional structure, stability, and conformational changes. The MD simulations clearly confirmed the stability of the three predicted TaNHX proteins over the entire simulation period. These findings collectively offer valuable insights into the expression patterns of *TaNHX* genes under salinity stress and their potential role in maintaining Na^+^ ion homeostasis. By elucidating the distinctive expression patterns and robust stability of TaNHX proteins, our study contributes significantly to the comprehension of the molecular mechanisms that underlie salt tolerance in wheat.

## 5 Conclusions

A comprehensive analysis of the *TaNHX* gene family in wheat was carried out in the present study. A total of 30 *TaNHX*s had been identified and phylogenetically divided into three subfamilies, as supported through highly conserved gene structure and motifs in addition to regulatory elements, revealing their significant role in wheat tolerance under salt stress. These thirty *TaNHX* genes were unevenly distributed among 18 chromosomes in wheat, except chromosomes 6A, 6B, and 6D, wherein no genes have been present. Collinearity analysis results confirmed that segmental duplication events had been essential for the expansion of *TaNHX* genes in the wheat genome and that certain *TaNHX* genes might also had been formed through gene duplication. Through qRT-PCR analysis, we observed that certain *TaNHX* genes in wheat showed increased expression under salt stress, with varying expression patterns at different time intervals in salt-tolerant and salt-sensitive cultivars of wheat. These findings emphasize the significance of the NHX family in wheat tolerance for salt stress response and offer a strong foundation for in addition exploration of wheat NHX genes.

## Data availability statement

The datasets presented in this study can be found in online repositories. The names of the repository/repositories and accession number(s) can be found in the article/**Supplementary Material**.

## Author contributions

PS: Conceptualization, Formal analysis, Investigation, Supervision, Writing – original draft, Writing – review & editing, Project administration. SM: Data curation, Formal analysis, Methodology, Software, Validation, Writing – original draft. BP: Data curation, Software, Validation, Writing – review & editing. GS: Funding acquisition, Writing – review & editing.

## Supplementary material

Supplementary Figure 1 Amino acid sequences of motifs in logos format.

Supplementary Figure 2 Interaction network of NHX family in wheat list of significant GO term (FDR <0.01) of nodes presented in network.

Supplementary Figure 3
*In-silico* expression analysis of NHX genes in various tissues under drought and heat stress.
