# Supplementary figures and images for: Genome-wide identification and expression analysis of the NHX gene family under salt stress in wheat (Triticum aestivum L)
Source: Front Plant Sci. 2023 Dec 4;14:1266699. doi: 10.3389/fpls.2023.1266699 (PMC10726055; doi:10.3389/fpls.2023.1266699)

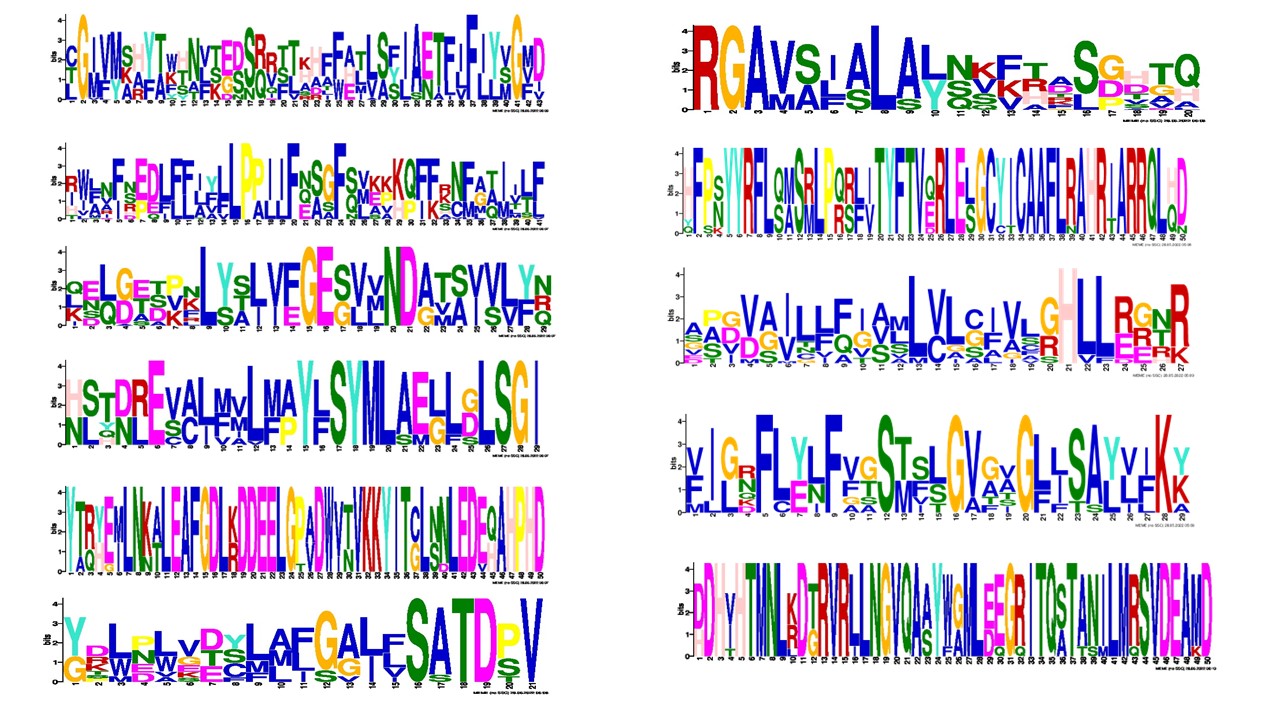

Supplement: Supplementary Figure 1 — Amino acid sequences of motifs in logos format. [file Image_1.jpeg]

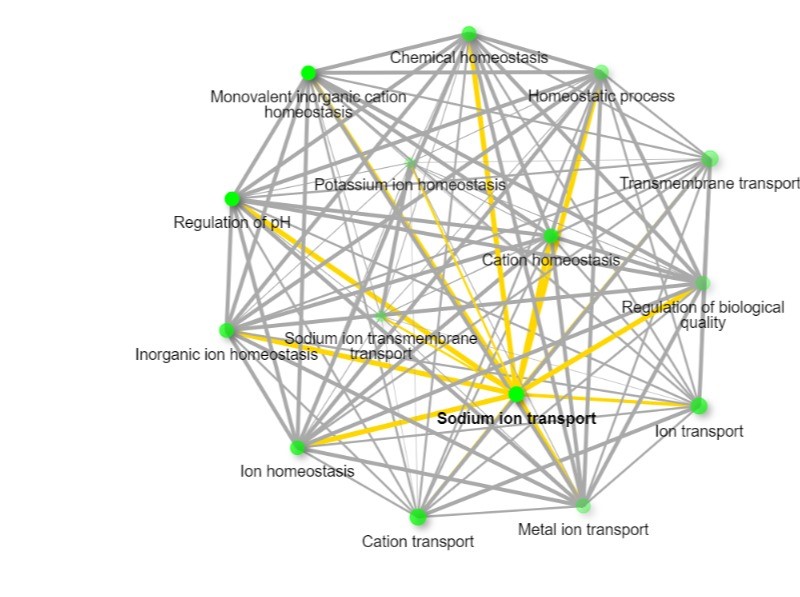

Supplement: Supplementary Figure 2 — Interaction network of NHX family in wheat list of significant GO term (FDR <0.01) of nodes presented in network. [file Image_2.jpeg]

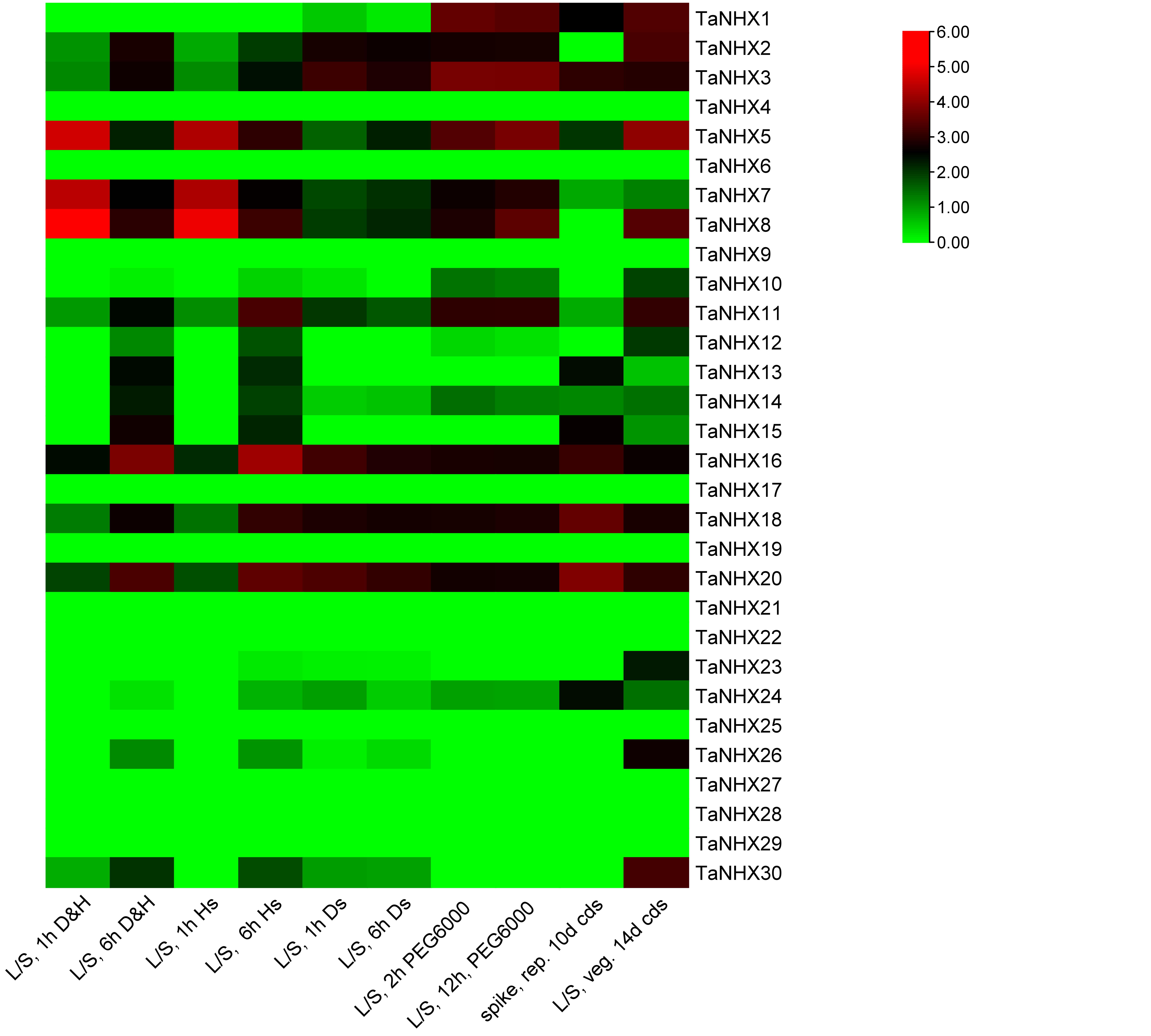

Supplement: Supplementary Figure 3 — In-silico expression analysis of NHX genes in various tissues under drought and heat stress. [file Image_3.jpeg]
